# Distributed Coding of Evidence Accumulation across the Mouse Brain Using Microcircuits with a Diversity of Timescales

**DOI:** 10.1523/ENEURO.0282-23.2023

**Published:** 2023-11-02

**Authors:** Elaheh Imani, Setayesh Radkani, Alireza Hashemi, Ahad Harati, Hamidreza Pourreza, Morteza Moazami Goudarzi

**Affiliations:** 1 Department of Computer Engineering, Ferdowsi University of Mashhad, Mashhad, 9177948974, Iran; 2 Brain and Cognitive Sciences, Massachusetts Institute of Technology, Cambridge, MA 02139; 3 The Decision Lab, Montreal, Quebec H4C 2C7, Canada

**Keywords:** accumulation mechanism, evidence accumulation, integration timescale, perceptual decision making

## Abstract

The gradual accumulation of noisy evidence for or against options is the main step in the perceptual decision-making process. Using brain-wide electrophysiological recording in mice (Steinmetz et al., 2019), we examined neural correlates of evidence accumulation across brain areas. We demonstrated that the neurons with drift-diffusion model (DDM)-like firing rate activity (i.e., evidence-sensitive ramping firing rate) were distributed across the brain. Exploring the underlying neural mechanism of evidence accumulation for the DDM-like neurons revealed different accumulation mechanisms (i.e., single and race) both within and across the brain areas. Our findings support the hypothesis that evidence accumulation is happening through multiple integration mechanisms in the brain. We further explored the timescale of the integration process in the single and race accumulator models. The results demonstrated that the accumulator microcircuits within each brain area had distinct properties in terms of their integration timescale, which were organized hierarchically across the brain. These findings support the existence of evidence accumulation over multiple timescales. Besides the variability of integration timescale across the brain, a heterogeneity of timescales was observed within each brain area as well. We demonstrated that this variability reflected the diversity of microcircuit parameters, such that accumulators with longer integration timescales had higher recurrent excitation strength.

## Significance Statement

In this paper, we characterized the perceptual decision-making process across the mouse brain. Our findings shed more light on the decision-making process by analyzing the brain-wide electrophysiological recording dataset. This paper contains a comprehensive analysis to characterize different aspects of the evidence accumulation process, including the distribution of accumulator-like neurons, the timescale of information integration, accumulation architecture, and the relationship between accumulators’ timescale and their integration circuit properties.

## Introduction

Decision-making, the process of choosing between options, is a fundamental cognitive function. Different types of decision-making, including perceptual (Gold and Shadlen, 2007) and value-based decision-making (Hunt et al., 2012), is thought to be characterized by a gradual accumulation of noisy evidence for or against options until a threshold is reached and a decision is made. The study of the evidence accumulation process started within cognitive psychology, where researchers explored sequential sampling models, i.e., the drift-diffusion model (DDM), using behavioral data (Ratcliff and McKoon, 2008). In these models, noisy information is accumulated over time from a starting point toward a decision boundary.

Later studies on the neural basis of decision-making developed computational models for the accumulation process using neurons showing signatures of the drift-diffusion model, referred to as DDM-like neurons (X.J. Wang, 2002; Mazurek et al., 2003). DDM-like neurons exhibit ramping-like firing rate activity modulated with stimulus coherency. These studies explored some brain regions containing DDM-like neurons, such as the posterior parietal cortex (PPC; Shadlen and Newsome, 2001; Roitman and Shadlen, 2002), frontal eye field (FEF; Kim and Shadlen, 1999; Ding and Gold, 2012), striatum (Ding and Gold, 2010), and superior colliculus (Horwitz and Newsome, 1999) in monkeys, as well as the frontal orienting field (FOF) and PPC (Hanks et al., 2015) in rats.

Although previous studies on the neural basis of decision-making explored a few brain regions showing the neural correlate of evidence accumulation, the distribution of DDM-like neurons across the brain is still unknown. Recent brain-wide electrophysiological and calcium imaging studies in mice revealed that neurons involved in decision-making are distributed across the brain (Steinmetz et al., 2019; Zatka-Haas et al., 2021). These findings motivated us to explore the existence of choice-selective neurons that have DDM-like firing rate activity across the brain. Similar to the drift-diffusion process, these neurons have ramping-like firing rates associated with the strength of stimulus evidence, such that stronger evidence levels lead to a faster ramping of firing rate and vice versa. However, these patterns of activity can be explained by different accumulation mechanisms, i.e., single (DDM) and dual accumulators (Bogacz et al., 2006). Although several accumulation models have been proposed in previous studies (Usher and McClelland, 2001; Mazurek et al., 2003; Machens et al., 2005; Wong and Wang, 2006), we examined the popular accumulator circuits (i.e., single and race accumulators) to characterize the underlying neural mechanism of evidence accumulation.

Moreover, the distributed coding of evidence accumulation suggests multiple timescales over this cognitive process (Chen et al., 2015). This property stems from the fact that each brain area exhibits a distinct timescale leading to a hierarchical organization that largely follows the anatomic hierarchy (Honey et al., 2012; Murray et al., 2014; Chen et al., 2015; Rossi-Pool et al., 2021; Pinto et al., 2022; Imani et al., 2023). As such, we used the brain-wide electrophysiological recording data recently published by Steinmetz et al. (2019) to investigate the distribution of DDM-like neurons and the underlying neural mechanisms of evidence accumulation across the brain. We demonstrated that evidence accumulation is a distributed process across the brain that is happening through multiple accumulation mechanisms. Our findings revealed that some areas are unilateral and strongly prefer the single accumulation mechanism. On the other hand, some areas are bilateral and contain subpopulations with both single and dual accumulation mechanisms. We further studied the timescale of integration using the simulated data from accumulator models across the brain. The results demonstrated that the accumulator microcircuits have distinct timescales, which were organized hierarchically across the brain, suggesting the existence of evidence accumulation over multiple timescales. Moreover, we observed a heterogeneity of integration timescales within each brain region, reflecting the diversity of recurrent connection strength of the accumulators. Our findings support the hypothesis that microcircuits with longer integration timescales have higher recurrent connection strength.

## Materials and Methods

### Behavioral task

**Figure 1.**
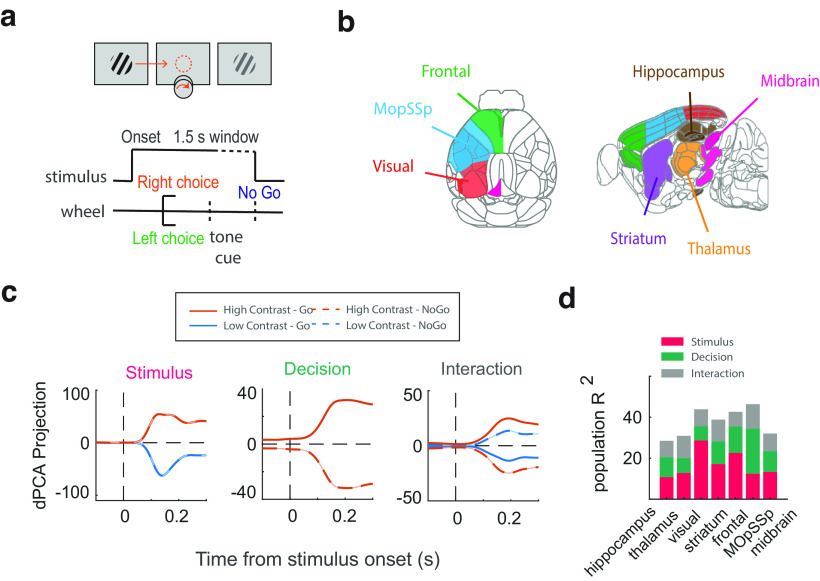
Decomposing brain-wide electrophysiological data into the task-related components using dPCA. ***a***, Task protocol. ***b***, Grouping the brain-wide electrophysiological data into the seven regions according to the Allen CCF adapted from previously published work (Steinmetz et al., 2019). ***c***, Projecting the population firing rate into the stimulus, decision, and interaction components. ***d***, *R*^2^ values of the reconstructed population neural activity using the task-related dPCA components.

We used a publicly available dataset published recently by Steinmetz et al. (2019). The dataset comprises behavioral and physiological data from ten mice over 39 sessions on a two-alternative unforced choice task. Mice sit on a plastic apparatus with their forepaws on a rotating wheel, surrounded by three computer monitors. At each trial that was started by briefly holding the wheel, visual stimuli (Gabor patch with σ 9° and 45° direction) with four grading levels were displayed on the right, left, both, or neither screen (Fig. 1*a*). The stimulus was presented in the mouse’s central monocular zones, and the animal did not need to move its head to perceive it.

10.1523/ENEURO.0282-23.2023.f1-1 Extended Data Figure 1-1 Separating neurons into the decision-selective and the stimulus-selective neurons. ***a***, Projection of the population neural activity into task-related components. ***b***, Clustering the neurons based on their stimulus-related and decision-related *R*^2^ values. ***c***, Performance of the stimulus and decision decoding using each group of neurons (stimulus, decision, and interaction). Shaded areas represent the 95% confidence interval. Download Figure 1-1, EPS file.

Mice earned water by turning the wheel to move the stimulus with the highest contrast to the center of the screen or by not turning the wheel if neither stimulus was displayed. Otherwise, they received a white noise sound for 1 s to indicate an improper wheel movement. Therefore, three types of trial outcomes (right turn, left turn, and no turn) leads to reward. After the stimulus presentation, a random delay interval of 0.5–1.2 s was considered, during which the mouse could freely turn the wheel without incentive. At the end of the interval, an auditory tone cue (8-kHz pure tone for 0.2 s) was played, at which point the visual stimulus position became coupled with the wheel movement.

### Neural recording

Recordings were made in the left hemisphere using the Neuropixel electrode arrays from ∼30,000 neurons in 42 brain areas in 39 sessions. Using the Neuropixel probes with the ability to record from multiple brain regions produced data simultaneously recorded from several regions in each session. The neural activity of the regions was divided into seven main groups according to the Allen Common Coordinate Framework (CCF) atlas (Q. Wang et al., 2020; Fig. 1*b*). We performed all the analyses on these groups of regions.

### Single-neuron decoding analysis

We performed the single neuron decoding using the area under the receiver operating characteristic (auROC) analysis. The auROC metric was initially employed to measure the neuron’s choice probability (CP) based on the Mann–Whitney *U* statistic (Britten et al., 1996). Using this nonparametric statistical method, we can measure the differences between spike count distributions of two conditions (or behavioral outputs) to examine whether the neuron’s firing rate is significantly greater than the other condition. According to the task design, the stimulus and choice encoding are highly correlated and cause the decoding analysis. To overcome this limitation, we used combined condition auROC analysis to compute stimulus selectivity, choice probability, detect probability (DP), and evidence selectivity. The trials were then divided into different groups according to the task conditions, and the weighted average of the auROC values across conditions was considered the final decoding result. For this analysis, the neuron’s spikes were binned at 0.005 s and smoothed using a causal half-Gaussian kernel with a SD of 0.02 s. We also z-scored the firing rate of the neurons by subtracting the mean and dividing by the SD calculated during the baseline period (−0.9 to −0.1 s, stimulus aligned) across all trials.

#### Stimulus selectivity

We computed the contra stimulus selectivity using the combined condition auROC metric. Accordingly, the trials were divided into 12 groups based on the different choice alternatives (right, left, NoGo) and stimulus contrast levels (0, 0.25, 0.5, 1) presented on the left screen. We then applied the Mann–Whitney *U* statistic to measure the stimulus selectivity by comparing the spike counts of a neuron within the trials with the right stimulus higher than zero with the trials having the right stimulus equal to zero. The final stimulus selectivity was measured using the weighted average across 12 conditions.

#### Choice probability

Using the combined condition auROC statistic, we tested whether the neurons encode the choice. To compensate for the effect of the stimulus conditions, we divided the trials into 12 groups based on different combinations of right and left stimulus contrast levels, ignoring equal contrast conditions. Within each condition, we used the Mann–Whitney *U* statistic to compare the spike count of the trials with right/left choice with another choice in a window ranging from −0.3 to 0.1 s (aligned with wheel movement). A weighted average was then used to compute the final choice probability over different conditions. The absolute deviation of auROC from the chance level was considered as the choice selectivity: CP=|auROC−0.5|.

#### Detect probability

We also measured how well the neural activity encodes whether or not the animal turned the wheel correctly and referred to this measurement as “detect probability” (Hashemi et al., 2018). Accordingly, the trials were divided into 12 groups based on the different combinations of the right and left stimulus contrast levels, excluding the conditions with equal contrast levels. We then measured whether the Hit (correctly turning the wheel) trials had greater neural activity than the Missed trials using the Mann–Whitney *U* statistic during the stimulus epoch (−0.1 to 0.3 s). The level of selectivity for this measurement was calculated as the deviation of auROC from the chance level: DP=auROC−0.5.

#### Evidence selectivity

**Figure 2.**
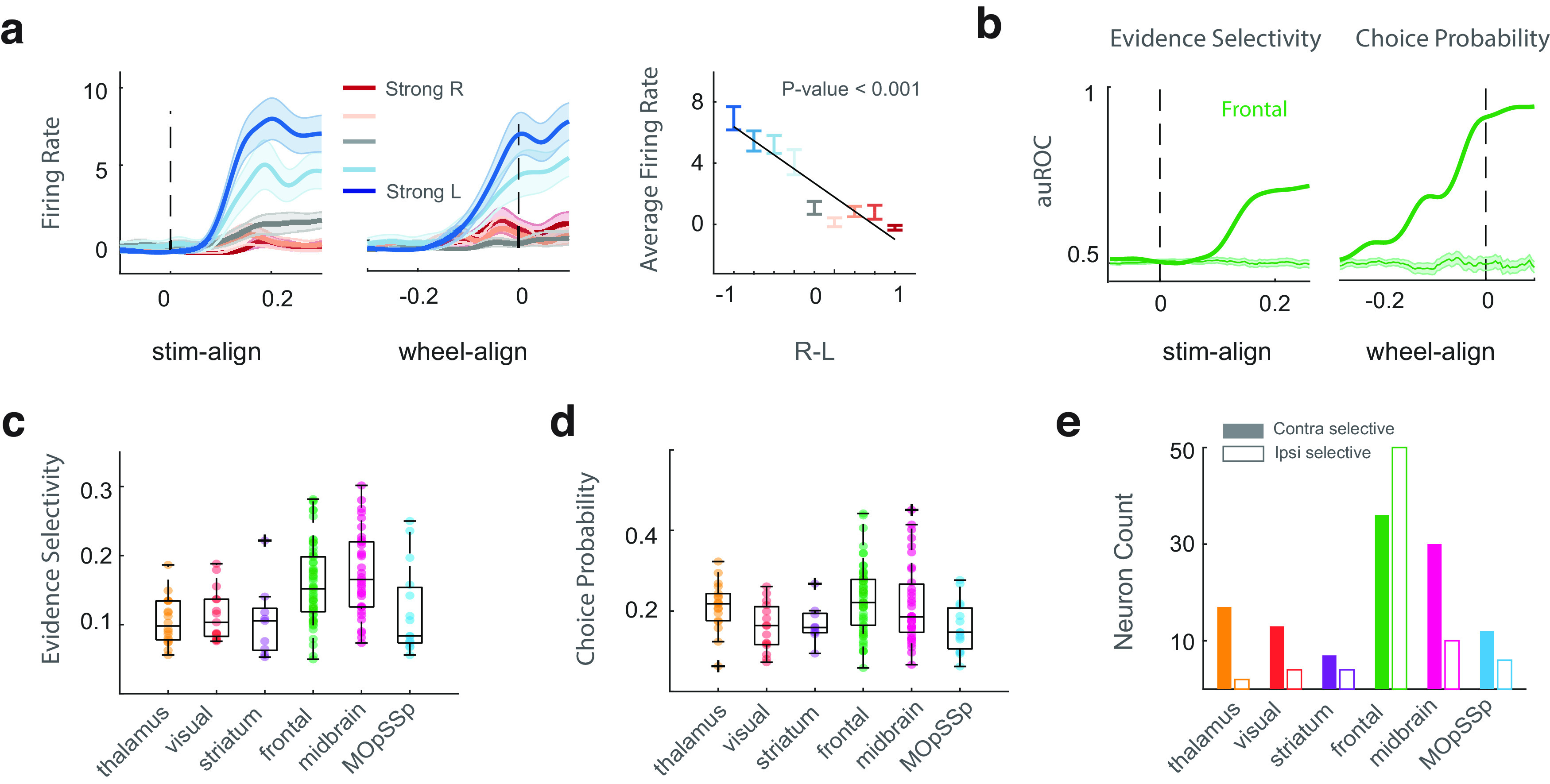
DDM-like neurons across the mouse brain. ***a***, Example neuron with DDM-like firing rate activity. The curves in the left panel represent neurons’ average firing rate activity across correct trials with a specific evidence level. The color strength indicates the strength of the evidence level, ranging from strong leftward to strong rightward. Shaded areas represent the 95% confidence interval. The right panel shows the linear relationship between the average firing rate of the neuron and the evidence levels using a general linear model. The colors represent the strength of the evidence level and the error bars indicate the 95% confidence interval. ***b***, Temporal evidence selectivity and choice probability for a sample neuron in the frontal region. Panels ***c*** and ***d*** are the maximum values of evidence selectivity and choice probability of the DDM-like neurons, respectively. ***e***, The total number of DDM-like neurons within each brain region (thalamus = 19, visual = 17, striatum = 11, frontal = 86, midbrain = 40, MOpSSp = 18). Filled and empty bars represent the number of neurons with contralateral and ipsilateral choice preferences, respectively.

We measured how a neuron can encode the evidence (difference of right and left stimulus contrast levels) and defined it as “evidence selectivity” (Fig 2*b*). The trials were divided into nine groups according to the number of evidence levels (ranging from −1 to 1 with a step size of 0.25). We then tested to see whether the group of trials with the higher evidence level had greater neural activity than all those groups with lower evidence. Final evidence selectivity was calculated by taking the weighted average of auROC values across eight group comparisons. Absolute deviation of auROC from the chance level was considered as the measure of evidence selectivity: ES=|auROC−0.5|.

10.1523/ENEURO.0282-23.2023.f2-1 Extended Data Figure 2-1 Distribution of DDM-like neurons across the brain. ***a***, Sample DDM-like neurons. The left panel represents the average firing rate activity of the neuron across trials with a specific evidence level. The strength of the color indicates the strength of the evidence level. Shaded areas represent the confidence interval. The right panels indicate the linear relationship between the average firing rate and the evidence levels using the general linear model. The error bars indicate the 95% confidence interval. ***b***, The number of DDM-like neurons across different brain areas. ***c***, Distribution of DDM-like across the brain. Download Figure 2-1, EPS file.

#### Significant auROC selectivity

We also performed the auROC analysis on the shuffled trial labels to identify significantly selective neurons. We created the distribution of the auROC on the shuffled trials by repeating the shuffling process 100 times. The selectivity of a neuron at time *t* was considered significant if the value of the true auROC was outside the confidence interval of the shuffled auROC values. We restricted our analysis to the time points with at least two significant neighbors to correct the multiple comparisons.

### Neuron latency

Evidence accumulation usually starts after a latency, mainly related to the visual encoding state (Roitman and Shadlen, 2002). In this study, we restricted the evidence accumulation analysis to the neural activity within the window starting at the end of latency until 50 ms after wheel movement. We used auROC analysis to compute latency which appears like the time of significant change in neural activity compared with the baseline activity.

Accordingly, the spike counts of the Go trials having reaction times within the range (0.15 to 0.5 s) were smoothed using a causal boxcar filter of size 100 ms during (−0.5 to 0.5 s) aligned to stimulus onset. We then computed the average firing rate across neurons within each trial, followed by the Mann–Whitney *U* statistic to compare the neural activity of trials within the stimulus (0 to 0.5 s) and a point in the baseline (−0.1 s) epochs. The significance level (*p*-value < 0.05) was employed to detect the samples with significant neural activity changes. We restricted our analysis to the significant points with at least two significant neighbors to correct for multiple comparisons. The latency was then selected as the first time point with significant changes in neural activity.

### Demixed principal component analysis (dPCA)

Most neurons, especially in the higher cortical areas, encode different types of task information and display a mixed selectivity (Kobak et al., 2016). This complexity in response selectivity of the neurons can conceal their expressed information. To overcome this limitation, we exploited the advantage of demixed principal component analysis (dPCA) to decompose the population neural activity into a few latent components, each capturing a specific aspect of the task (Kobak et al., 2016). The resulting dPCA subspace captures most variation in the data and decouples different task-related components.

According to the dPCA analysis, we prepared a matrix $X_{N\times 4\times 2\times T}$ containing marginalized activity of N neurons over four stimulus contrast levels (1, 0.5, 0.25, 0) and two decision alternatives (Hit and Missed) during T time points within the stimulus epoch (−0.1 to 0.3 s). We excluded the neurons having an average total spike count lower than one during the reaction time boundary (from stimulus onset until wheel movement). We first divided trials into four groups to construct the matrix based on the contralateral stimulus contrast levels. Within each group, the average trial activity of each neuron was then computed based on whether or not the animal turned the wheel. The dPCA algorithm was applied to the neural population matrix to construct a latent subspace with 20 task-related principal components. The resulting components characterized the decision ( $X_{dt}$), stimulus ( $X_{st}$), stimulus-decision interaction ( $X_{sdt}$), and condition-independent ( $X_{t}$) information by estimating task-specific decoders $F_{\alpha }$ and encoders $D_{\alpha }$ using the following loss function (Kobak et al., 2016):

(1) $$L_{dPCA}=\underset{\alpha }{\sum }\|X_{\alpha }-F_{\alpha }D_{\alpha }X{\|}^{2},\alpha \in \left\{t,st,dt,sdt\right\}.$$

We computed the explained variances $R_{\alpha }^{2}$ (*R*^2^) of the neurons by projecting the neural activity to the task-specific principal components using the decoder matrices $D_{\alpha }$ and reconstructing the neural activity with the decoder matrices $F_{\alpha }$ as follows:

$$\bar{X}=\frac{1}{4}\frac{1}{2}\frac{1}{T}\overset{4}{\underset{s=1}{\sum }}\overset{2}{\underset{d=1}{\sum }}\overset{T}{\underset{t=1}{\sum }}X_{sdt}$$

(2) $$R_{\alpha }^{2}=1-\frac{\sum_{s=1}^{4}\sum_{d=1}^{2}\sum_{t=1}^{T}{\left(F_{\alpha }D_{\alpha }X_{sdt}-X_{sdt}\right)}^{2}}{\sum_{s=1}^{4}\sum_{d=1}^{2}\sum_{t=1}^{T}{\left(X_{sdt}-\bar{X}\right)}^{2}},\alpha \in \left\{st,dt\right\}$$

We then separated the neurons into stimulus, decision, and interaction groups within each brain region using their task-related *R*^2^ values ( $R_{st}^{2}$, $R_{dt}^{2}$) and fuzzy C-means clustering algorithm (Bezdek, 2013). We excluded the neurons within the stimulus and interaction clusters from further analysis.

### Integration timescale

We measured the integration timescale of the subpopulations using the spike count autocorrelation structure of the simulated neural activity. Accordingly, we simulated fixed-length trials of duration 200 ms for each subpopulation using the preferred (single or race) accumulator model during a 50-times sampling process. We then estimated the timescale of simulated neurons within each sample set of trials and considered the average timescale across the 50 samples as the final timescale for the subpopulation. To estimate the timescale of simulated neurons at each sampling iteration, we computed the Pearson’s correlation of binned spike counts between each pair of time bins iΔ and jΔ( j>i, Δ=0.025s) across trials. The resulting autocorrelation values follow an exponential decay which can be explained using the following equation (Murray et al., 2014):

(3) $$R(k\Delta )=A\times (exp(-\frac{k\Delta }{\tau })+B),$$

where A is the amplitude, B indicates the contribution of timescales longer than the observation window, kΔ is the time lag, and τ denotes the timescale.

We fitted Equation 3 to the combined autocorrelation structure of the simulated neurons within each subpopulation using the Levenberg–Marquardt method. The time lag with the greatest autocorrelation reduction was selected as the starting point for overcoming the negative adaptation (Murray et al., 2014). We tried five different initial parameter values to select the best model having the lowest mean square error (MSE) value. We eventually computed the average of timescales across 50 sets of simulated trials.

Similarly, the global population-level timescale of each brain region was estimated based on the combined autocorrelation structure of the simulated neurons. We bootstrapped subpopulations within each brain area 100 times to compute the confidence interval of the population-level timescales. We further applied a Wilcoxon rank-sum test on the bootstrapped samples to test for significant differences between regions.

### Recurrent switching linear dynamical system (rSLDS)

**Figure 3.**
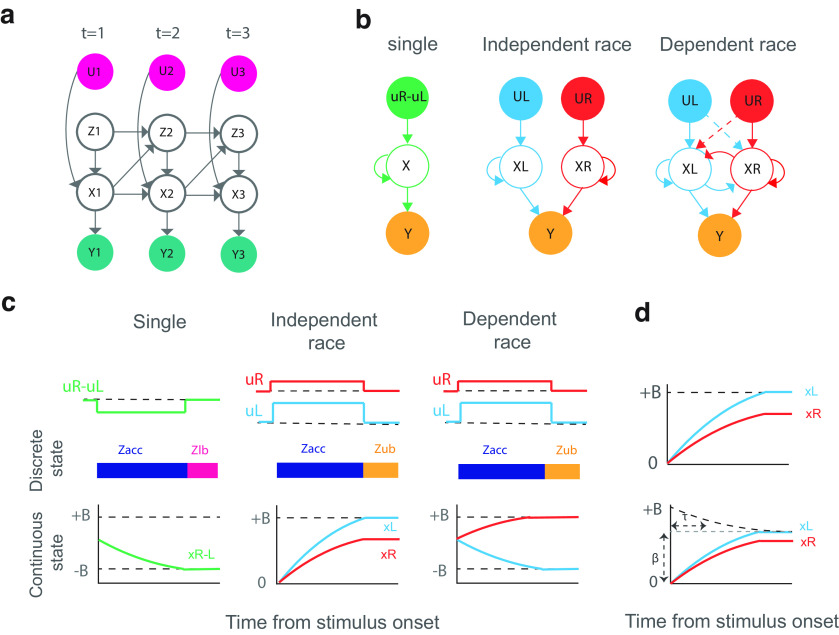
Reformulating the recurrent switching linear dynamical system (rSLDS) framework to the single and dual (independent/dependent race) accumulation mechanisms. ***a***, Schematic of the rSLDS containing the hidden discrete variable Z, hidden continuous variable X, and observed variables U related to the stimulus strength and neuron spike data Y. ***b***, Single, independent race, and dependent race accumulator models implemented in rSLDS. ***c***, Discrete and continuous states of the accumulation mechanisms within sample trials. ***d***, Constant (top row) and collapsing (bottom row) decision boundaries. The collapsing boundary contains two parameters β and τ, for the boundary offset and the rate of exponential decay.

We employed a general framework proposed by Zoltowski et al. (2020) for modeling the evidence accumulation process. Different evidence accumulation models are formulated in this framework as a recurrent switching linear dynamical system (rSLDS). The rSLDS contains multiple discrete states $z_{t}\in \left\{1,...,K\right\}$ and each state is associated with specific linear dynamics (Fig. 3*a*) as follows:

(4) $$x_{t}∼N(A^{z_{t}}x_{t-1}+V^{z_{t}}u_{t}+b^{z_{t}},Q^{z_{t}}),$$

where $x_{t}\in R^{D}$ is the continuous state, $u_{t}\in R^{M}$ represents input streams, $Q^{z_{t}}\in R^{D\times D}$ is the noise covariance matrix, and the matrices $A^{z_{t}}\in R^{D\times D}$ and $V^{z_{t}}\in R^{D\times M}$, and vector $b^{z_{t}}\in R^{D}$ denote the state-specific dynamic parameters. Transition probabilities between discrete states are parameterized as follows:

(5) $$p\left(z_{t-1},x_{t-1}\right)\propto exp\left\{\left(R_{z_{t-1}}x_{t-1}+r_{z_{t-1}}\right)\right\},$$

where $R_{z_{t-1}}\in R^{K\times D}$ and $r_{z_{t-1}}\in R^{K}$ parameterize the influence of the continuous state on the discrete state transitions. The observation model was used to map the continuous latent variables $x_{t}$ into the overserved variable $y_{t}$ using the Poisson distribution of a generalized linear model as follows:

(6) $$y_{t}∼Poisson\left(f\left(Cx_{t}+d\right)\Delta_{t}\right),$$

where f(x)=log(1+exp(x)) is the Softplus function, and $\Delta_{t}$ denotes the size of time bins. The weight parameter $C\in R^{D\times N}$ was used to map the latent variable $x_{t}\in R^{D}$ into the activity of N neurons $y_{t}$. However, the offset parameter $d\in R^{D}$ in the observation model is shared across the neurons.

#### Single accumulator

A single accumulator model, which is commonly referred to as the drift-diffusion model (DDM), is described with a single decision variable that accumulates the differences in the input streams (Bogacz et al., 2006). This accumulation mechanism has two decision boundaries, one for each choice alternative. When the decision variable reaches one of the boundaries, the decision is made.

To reformulate the rSLDS framework to a single accumulator, we considered three discrete states for the accumulation ( $z_{t}=acc$) phase, right wheel movement ( $z_{t}=rwm$), and left wheel movement ( $z_{t}=lwm$; Fig. 3*c*). During the evidence accumulation state, the one-dimensional continuous variable $x_{t}\in R^{1}$ accumulates the differences between right and left input streams $u_{t}\in R^{1}$. The state transition was also parameterized such that when the continuous variable $x_{t}$ reaches one of the decision boundaries (±B), the discrete state switches from the accumulation state ( $z_{t}=acc)$ to the right wheel movement ( $z_{t}=rwm$) or left wheel movement ( $z_{t}=lwm$) states. Therefore, the transition parameters were set as follows:

(7) $$R_{z_{t-1}}=\left[\begin{array}{c} 0\\ 1\\ -1 \end{array}\right],r_{z_{t-1}}=\left[\begin{array}{c} 0\\ -B\\ -B \end{array}\right],\gamma =1,B=1.$$

According to the settings, increasing the value of $x_{t}$ toward B leads to an increase in the probability of transition from the $z_{t}=acc$ to $z_{t}=rwm$. On the other hand, decreasing the value of $x_{t}$ toward -B, increases the probability of transition to $z_{t}=lwm$.

In Equation 4, the term A∈R, denotes the recurrent connection strength, and the term V∈R determines the weight of the received input stream (Fig. 3*b*). We excluded the term b∈R from our analysis. In the accumulation state ( $z_{t}=acc$), we only trained the term $A^{acc}$ and the noise $Q^{acc}$, and set the term $V^{acc}$ constant. In other states ( $z_{t}=rwm,lwm$), we just trained the noise variance Q and considered $A^{rwm}=A^{lwm}=1$ and $V^{rwm}=V^{lwm}=0$. We tried different initial values for the parameters $A^{acc}\in \left\{0.95,1\right\},V^{acc}\in \left\{0.01,0.02,0.03,0.04,0.05\right\},Q\in \left\{0.005,0.01\right\}$ to select the best combination of parameters that produced maximum log-likelihood.

#### Independent race accumulator

An independent race accumulator model contains two integrators that accumulate the relative or absolute input streams supporting each choice alternative (Bogacz et al., 2006). In this accumulation mechanism, a decision is made favoring the integrator that reaches the decision boundary sooner. To reformulate the rSLDS into an independent race accumulator mechanism, we considered a two-dimensional continuous variable $x_{t}\in R^{2}$ for two accumulators. These variables accumulated the absolute right/left input streams $u_{t}\in R^{2}$ independently. We set the parameters of the dynamic model $\left(A_{acc},V_{acc},Q_{acc}\right)$ to be diagonal such that the decision variables integrate the input streams independently. Similar to the single accumulator, we considered three discrete states for the accumulation ( $z_{t}=acc$) phase, right wheel movement ( $z_{t}=rwm$), and left wheel movement ( $z_{t}=lwm$; Fig. 3*c*).

We also set the transition parameters such that the probability of switching from the accumulation state to one of the wheel movement states increases by approaching $x_{t}$ to decision boundary *B*. Accordingly, the transition parameters were set as follows:

(8) $$R_{z_{t-1}}=\left[\begin{array}{cc} 0 & 0\\ 1 & 0\\ 0 & 1 \end{array}\right],r_{z_{t-1}}=\left[\begin{array}{c} 0\\ -B\\ -B \end{array}\right],\gamma =1,B=1.$$

In Equation 4, on-diagonal values in matrix $A\in R^{2}$ determines the excitatory connection strength for two accumulators. The on-diagonal values in matrix $V\in R^{2}$ also denotes the weight of the received input stream for each accumulator variable (Fig. 3*b*). Similar to the single accumulator mechanism, we excluded term *b* from our analysis. In the accumulation state ( $z_{t}=acc$), we only trained matrices $A^{acc}$ and noise $Q^{acc}$, and set the matrix $V^{acc}$ constant. In other states ( $z_{t}=rwm,lwm$), we just trained the noise covariance matrix Q and considered $A^{rwm}=A^{lwm}=I$ and $V^{rwm}=V^{lwm}=0_{2,2}$. We tried different initial values for the on-diagonal values of matrices $A^{acc}\in \left\{0.95,1\right\},V^{acc}\in \left\{0.01,0.02,0.03\right\},Q\in \left\{0.005,0.01\right\}$ to select the best combinations of parameters that produced maximum log-likelihood.

#### Dependent race accumulator

Dependent race models are a more general form of dual accumulators containing mutual (Usher and McClelland, 2001; Machens et al., 2005; Wong and Wang, 2006) and feedforward connections (Purcell et al., 2010; Palmeri et al., 2015). In these models, each decision variable accumulates input streams supporting each choice alternative and the decision is made favoring the integrator that reaches the respective decision boundary sooner.

Similar to the independent race model, we consider a two-dimensional continuous variable $x_{t}\in R^{2}$ to reformulate rSLDS into the dependent race accumulator. To model the mutual and feedforward connections, we considered parameters in the dynamic model $\left(A_{acc},V_{acc},Q_{acc}\right)$ to be fully connected rather than diagonal. Because of the negative and positive decision boundaries in this model, we considered five discrete states for the accumulation ( $z_{t}=acc$) phase, positive/negative right wheel movement ( $z_{t}=\text{p}rwm,z_{t}=\text{n}rwm$), and positive/negative left wheel movement ( $z_{t}=\text{p}lwm,z_{t}=\text{n}lwm$).

The transition parameters are set such that the probability of switching from the accumulation state to the right or left wheel movement states increases by approaching $x_{t}$ to each of the decision boundaries ±*B*. Accordingly, the transition parameters were set as follows:

(9) $$R=\left[\begin{array}{cc} 0 & 0\\ 1 & 0\\ \begin{array}{c} -1\\ 0\\ 0 \end{array} & \begin{array}{c} 0\\ 1\\ -1 \end{array} \end{array}\right],r=\left[\begin{array}{c} 0\\ -B\\ \begin{array}{c} -B\\ -B\\ -B \end{array} \end{array}\right],\gamma =1,B=1.$$

In the accumulation state ( $z_{t}=acc$), we only trained matrices $A^{acc}$ and noise $Q^{acc}$, and set the matrix $V^{acc}$ constant. In the other four states, we just trained the noise covariance matrices Q and considered matrices A and V to be the identity and null matrices, respectively. We tried different initial values for the on-diagonal parameters $A^{acc}\in \left\{0.95,1\right\},V^{acc}\in \left\{0.01,0.02,0.03\right\},Q\in \left\{0.01\right\}$ and off-diagonal parameters $A^{acc}\in \left\{-0.05\right\},V^{acc}\in \left\{-0.01,-0.02,-0.03\right\}$ to select the best combinations of parameters that produced maximum log-likelihood.

#### Collapsing boundary

In the accumulators with the collapsing boundary, less evidence is required to reach the boundary as time passes so that the boundaries collapse toward the center (Fig. 3*d*). This mechanism is much like the urgency signal, magnifying the evidence as time passes (Ratcliff et al., 2016). Besides the constant decision boundaries, we also evaluated the collapsing boundary in single and dual accumulators.

In the rSLDS framework, we can reformulate Equation 5 to implement the linear collapsing boundary for a single accumulator as follows (Zoltowski et al., 2020):

(10) $$p\left(z_{t-1},x_{t-1}\right)\propto exp\left\{\gamma \left(R_{z_{t-1}}x_{t-1}+r_{z_{t-1}}+W{\bar{u}}_{t}\right)\right\},W=\left[\begin{array}{cc} 0 & 0\\ 0 & \beta \\ 0 & \beta \end{array}\right],{\bar{u}}_{t}=\left[\begin{array}{c} u_{t}\\ t \end{array}\right].$$

Where $W{\bar{u}}_{t}$ is the linear function of time points *t* and vector ${\bar{u}}_{t}$ contains the input streams and the current time. This equation describes a linear collapsing boundary with the rate of β. We need to add another column to the matrix V in Equation 4 and set it to zero with this new formulation. We further modified Equation 9 to formulate a nonlinear collapsing boundary for a single accumulator as follows:

(11) $$p\left(z_{t-1},x_{t-1}\right)\propto exp\left\{\gamma \left(Rx_{t-1}+r+Wf\left({\bar{u}}_{t}\right)\right)\right\}$$

$$f\left(t\right)=\beta +(1-\beta )\times \text{exp}(\frac{-t}{\tau })$$

$$W=\left[\begin{array}{cc} 0 & 0\\ 0 & -B\\ 0 & -B \end{array}\right],R=\left[\begin{array}{c} 0\\ 1\\ -1 \end{array}\right],r=\left[\begin{array}{c} 0\\ 0\\ 0 \end{array}\right],{\bar{u}}_{t}=\left[\begin{array}{c} u_{t}\\ t \end{array}\right].$$

Where β denotes the boundary offset and τ describes the decay rate of the exponential function. We can control the collapsing rate with these two parameters (Fig. 3*d*). To implement the collapsing boundary for the independent and dependent race accumulators, we set the parameters of the transition model as Equations 12 and 13, respectively:

(12) $$W=\left[\begin{array}{ccc} 0 & 0 & 0\\ 0 & 0 & -B\\ 0 & 0 & -B \end{array}\right],R=\left[\begin{array}{cc} 0 & 0\\ 1 & 0\\ 0 & 1 \end{array}\right],r=\left[\begin{array}{c} 0\\ 0\\ 0 \end{array}\right],{\bar{u}}_{t}=\left[\begin{array}{c} u_{t}\\ t \end{array}\right]$$

(13) $$W=\left[\begin{array}{ccc} 0 & 0 & 0\\ 0 & 0 & -B\\ \begin{array}{c} 0\\ 0\\ 0 \end{array} & \begin{array}{c} 0\\ 0\\ 0 \end{array} & \begin{array}{c} -B\\ -B\\ -B \end{array} \end{array}\right],R=\left[\begin{array}{cc} 0 & 0\\ 1 & 0\\ \begin{array}{c} -1\\ 0\\ 0 \end{array} & \begin{array}{c} 0\\ 1\\ -1 \end{array} \end{array}\right],r=\left[\begin{array}{c} 0\\ 0\\ \begin{array}{c} 0\\ 0\\ 0 \end{array} \end{array}\right],{\bar{u}}_{t}=\left[\begin{array}{c} u_{t}\\ t \end{array}\right].$$

We tried different initial values for β∈{0.3,0.4,0.5} and τ∈{50,100} to select the best parameter which leads to the maximum log-likelihood.

#### Model fitting

We fit the accumulator models to the subpopulations of neurons within the brain regions at each session. Accordingly, subpopulations were generated by sampling four DDM-like neurons without replacement within each brain area. To improve the performance of modeling, we excluded trials according to the stimulus and reaction time criteria. Accordingly, trials with equal contrast levels (Right = Left) were excluded because of the random behavioral output of mice during these trials. We further focused our analysis on the trials with reaction times longer than 150 ms and shorter than 500 ms.

To model the evidence accumulation process, we did not consider fixed-length trials. Given that the perceptual decision-making process comprises different cognitive stages (visual encoding, evidence accumulation, and action execution; Mazurek et al., 2003), we excluded the neural activity corresponding to the visual encoding phase (Roitman and Shadlen, 2002). The remaining samples before wheel movement are considered as the evidence accumulation phase. We also included the neural activity from the 50 ms postwheel movement period. This is because of considering multiple discrete states (i.e., accumulation and right/left wheel movement phases) to reformulate the recurrent switching linear dynamical system (rSLDS) into different accumulators. According to these settings, the continuous variables evolve in the accumulation state and switch to the right/left wheel movement state by reaching the corresponding decision boundary.

Zoltowski et al. (2020) introduced a variational Laplace-EM algorithm to estimate the model parameters. Briefly, the posterior over the discrete and continuous states were calculated using variational and Laplace approximations. The model parameters were also updated by sampling from the discrete and continuous posteriors followed by an expectation-maximization (EM) approach (Zoltowski et al., 2020).

#### Model goodness of fit

##### Akaike Information Criterion (AIC)

We compared the model fitting to the data using the Akaike Information Criterion (AIC) goodness of fit, which is defined as follows (Anderson and Burnham, 2004):

(14) $$AIC=2k-2E_{\theta |y_{t}}\left[logp\left(x_{t},z_{t},\theta \right)\right].$$

Where *k* is the number of free parameters in the model and the expectation term $E_{\theta |y_{t}}$ can be estimated by sampling the fitted model 100 times as follows:

(15) Eθ|yt[log(yt|xt,zt,θ)]=1s∑s=1100Eq(x)q(z)[log p(yt|xt,zt,θs)],

where $\theta^{s}$ denotes sampling of the model using trained parameters. To compute the log-likelihood, we need to marginalize the hidden variables *x* and *z*. Accordingly, we sampled from the estimated posterior probabilities [ q(x) and q(z)] to compute the sample-based expectation over these two variables (Zoltowski et al., 2020). AIC measurement contains a penalty term for the number of parameters which is a correction for how much the model with *k* parameters will increase the log-likelihood.

##### R^2^

We also measured how well a model can explain the data using the *R*^2^ explained variance. Accordingly, we simulated the spike counts from each model 100 times for each trial. The firing rate of the real and simulated spike counts of subpopulations was computed using a causal boxcar filter of size 50 ms, and the average firing rate of trials within each evidence level (right contrast level-left contrast level) was computed. We then used the *R*^2^ explained variance metric on the subpopulations as follows (Latimer et al., 2015):

$$R^{2}=1-\frac{\sum_{e\in S_{e}}\sum_{t\in S_{t}}{\left(f_{ne}\left(t\right)-f_{me}\left(t\right)\right)}^{2}}{\sum_{e\in S_{e}}\sum_{t\in S_{t}}{\left(f_{ne}\left(t\right)-\bar{f_{ne}}\right)}^{2}}$$

(16) $$\bar{f_{ne}}=\frac{1}{9}\frac{1}{T}\underset{e\in S_{e}}{\sum }\underset{t\in S_{t}}{\sum }f_{ne}\left(t\right),$$

where *e* is the evidence level from the set of evidence $S_{e}$, terms $f_{ne}$ and $f_{me}$ represent the average firing rate of the data and simulated spike counts across the trials with evidence level *e,* respectively. Set $S_{t}$ denotes the time points within the window from the latency until the median reaction time of the session. The Term $\bar{f_{ne}}$ is the average firing rate of the data over all time points and coherence levels. $R^{2}$ = 1 demonstrates that the model firing rate perfectly matches the data, and lower values correspond to the worst fit.

#### Model comparison

The preferred accumulator type among the single and race accumulators is selected using the AIC difference approach. According to this approach, the AIC values are rescaled as follows:

(17) $${\text{Δ}}_{i}=AIC_{i}-AIC_{min}.$$

**Figure 4.**
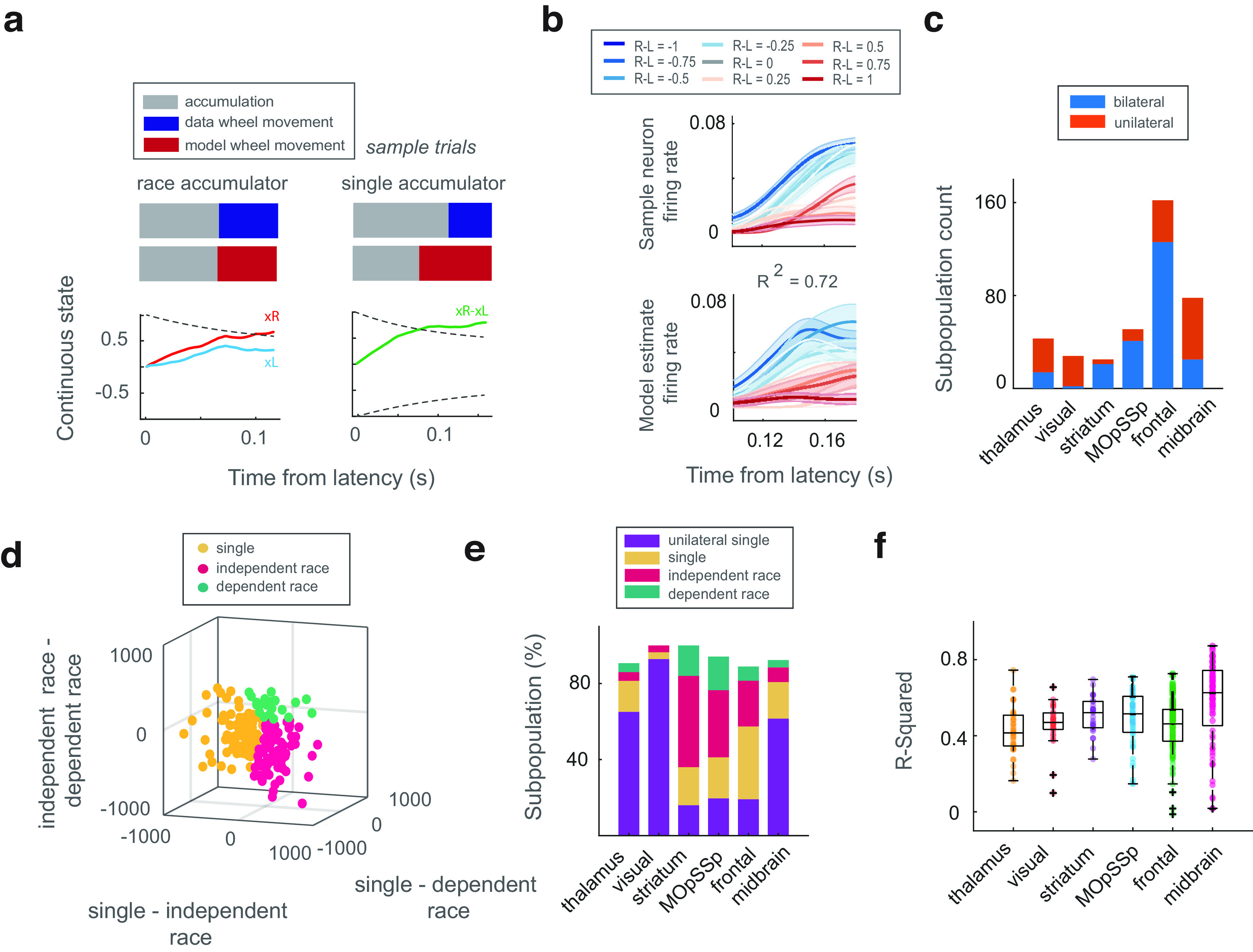
Evaluation of the single and race accumulator models. ***a***, The discrete state switches to the right choice state when the continuous variable reaches the collapsing boundary. ***b***, The firing rate of the sample neuron and the fitted single accumulator model. The explained variance (*R*^2^) between the data and model firing rate is depicted above the figure. The colors indicate the strength of the evidence level. ***c***, The number of bilateral and unilateral subpopulations within the brain regions. ***d***, Model comparison using the AIC difference approach. Each axis demonstrates the paired AIC difference. The best model is the one with a lower AIC value than others. The colors indicate the best accumulator model. ***e***, The percentage of bilateral and unilateral subpopulations preferring single, independent race, and dependent race accumulators. ***f***, Explained variance (*R*^2^) values for each brain region’s bilateral and unilateral subpopulations. *R*^2^ values were computed between data and the best model selected using the AIC difference for the bilateral subpopulations. For the unilateral subpopulations, this metric was computed using the single accumulator.

Where $AIC_{min}$ is the minimum of AIC values among the single and race accumulators for a specific subpopulation. According to this transformation, the best model has ${\text{Δ}}_{i}=0$ and other models have positive ${\text{Δ}}_{i}$ values. To select the best model we set the supporting threshold of 10 (Anderson and Burnham, 2004; Latimer et al., 2015). Accordingly, we excluded subpopulations having more than one model with an AIC difference ${\text{Δ}}_{i}<10$ from our further analysis. To visualize the preferred models, we computed the paired AIC differences ( ${\text{Δ}}_{si}=AIC_{single}-AIC_{independent\;race}$, ${\text{Δ}}_{sd}=AIC_{single}-AIC_{dependent\;race}$, ${\text{Δ}}_{id}=AIC_{independent\;race}-AIC_{dependent\;race}$). The preferred model is the one with a lower AIC value than other models resulting in negative AIC differences ${\text{Δ}}_{si}$ and ${\text{Δ}}_{sd}$ for single models and positive AIC differences ${\text{Δ}}_{sd}$ and ${\text{Δ}}_{id}$ for dependent race models. Similarly, subpopulations with independent race preference shave negative ${\text{Δ}}_{id}$ and positive ${\text{Δ}}_{si}$ values (Fig. 4*d*).

10.1523/ENEURO.0282-23.2023.f4-1 Extended Data Figure 4-1 Results of the accumulator fitting. Data and model firing rate of sample neurons and their corresponding explained variance (*R*^2^) value. The distribution of *R*^2^ values for each neuron was generated by sampling the accumulator model 100 times. The curves represent the average firing rate activity of the neuron across trials with a specific evidence level. The strength of the color indicates the strength of the evidence level. Shaded areas represent the confidence interval. ***b***, The proportion of bilateral subpopulations preferring single and race accumulators. ***c***, The percentage of the single and race accumulators among the combination of unilateral and bilateral subpopulations. Marker *** represents the *p*-value < 0.001 in the sign test. *p*-values were corrected by the Bonferroni multiple comparison correction. Download Figure 4-1, EPS file.

### Data processing

All analyses were conducted using customized MATLAB and Python code. Statistical analyses and fuzzy C-means clustering were performed using MATLAB toolboxes. Decomposing neural activity into different task-related variables was conducted using the open-source dPCA toolbox (Kobak et al., 2016). The accumulator analysis was performed using the recurrent switching linear dynamical system (rSLDS) toolbox (Zoltowski et al., 2020), which was customized by the authors.

### Data availability

All neural and behavioral data analyzed in this study are available at https://figshare.com/articles/steinmetz/9598406.

### Code accessibility

The code described in this paper is freely available online at the GitHub repository (https://github.com/ElahehImani/NeuralCorrelateEvidenceAcc).

## Results

### Distributed evidence accumulation across the mice’s brain

To investigate whether or not the evidence accumulation process is distributed across the brain, we used the brain-wide neural recording in mice during a visual discrimination task (Steinmetz et al., 2019). In each trial, a visual stimulus of varying contrast (Gabor patch with σ 9° and 45° direction) appeared on the right, left, both, or neither side screens. To get a reward, the mice had to turn the wheel to move the stimulus with the higher contrast into the center screen (Fig. 1*a*). During the visual discrimination task, the neural activity of ∼30,000 neurons in 42 brain areas was recorded using Neuropixel probes. We focused our analysis on the seven groups of brain areas demonstrated in Table 1, Extended Data Table 1-1, and Figure 1*b*, according to the Allen Common Coordinate Framework (CCF; X.J. Wang et al., 2020).

**Table 1:** Brain regions within each group of areas according to the Allen CCF

Group name	Regions within each group
Hippocampus	POST, SUB, DG, CA1, CA3
Thalamus	LP, LD, RT, MD, MG, LGd, VPM, VPL, PO, POL
Visual	VISp, VISrl, VISam, VISpm, VISl, VISa
Striatum	CP, GPe, ACB, LS
Frontal	MOs, ACA, PL, ILA, ORB
MOpSSp	MOp, SSp
Midbrain	MRN, SCm, SCs, APN, PAG, SNr

10.1523/ENEURO.0282-23.2023.tab1-1 Extended Data Table 1-1 The full name and acronym of brain regions within each group of areas according to the Allen CCF. Download Table 1-1, DOC file.

To detect the neurons with DDM-like firing rate activity, we first determined the choice-selective neurons within each group of regions. Preliminary analyses showed that most neurons simultaneously encode different task variables, especially in higher cortical areas. Therefore, we first used demixed principal component analysis (dPCA; Kobak et al., 2016) to decompose the population neural activity into a few principal components representing specific task variables (Fig. 1*c*). We then determined whether a neuron responded more strongly to the stimulus or decision by measuring the reconstructed neural activity’s explained variance (*R*^2^) using each set of stimulus and decision-related components (Extended Data Fig. 1-1*a*). The results revealed that neurons across the brain regions belong to one of three clusters: those best represented by the stimulus-related components, the decision-related components, or their interaction components (Fig. 1*d*; Extended Data Fig. 1-1*b*). We excluded the hippocampus region from further analyses because of poor performance in the clustering analysis.

We evaluated dPCA results using the standard auROC metric to measure how well a neuron encodes the stimulus or decision variables. This metric is commonly used to calculate the differences between spike count distributions across different conditions (Britten et al., 1996). There is a strong correlation between the stimulus and animal choice by design. So, we used the combined condition auROC metric to reduce the effect of other task variables on the decoding performance (see Materials and Methods). For the stimulus decoding, we measured the differences between the spike count distribution of trials with contralateral stimulus higher than zero and trials with zero contra stimulus contrast level for all 12 conditions.

Similarly, decision decoding was evaluated by measuring the differences between Hit and Missed trials within 12 conditions referred to as “detect probability” (DP; Hashemi et al., 2018). Our results showed that the stimulus-selective neurons detected by dPCA, indeed encoded the stimulus more strongly than the decision. Similarly, the decision-selective neurons encoded the decision better than the stimulus (Extended Data Fig. 1-1*c*).

Finally, we found the DDM-like neurons within the decision-related clusters across the brain. Previous studies on the neural basis of evidence accumulation have discovered that DDM-like neurons in the posterior parietal cortex (LIP area) had a ramping-like firing rate activity associated with the strength of a motion stimulus (Shadlen and Newsome, 2001; Roitman and Shadlen, 2002). Similar properties were also found in the mouse’s PPC (Hanks et al., 2015) and anterior dorsal striatum (ADS) in rats (Yartsev et al., 2018). According to the properties of DDM-like neurons, we found the choice-selective neurons that additionally encoded the strength of the input evidence (difference between Right and Left stimulus contrasts). We used the combined condition auROC metric to measure each neuron’s choice probability (CP) and evidence selectivity. Accordingly, we calculated the differences between trials with right and left choices within 12 groups to measure the CP. For measuring evidence selectivity, we evaluated whether or not the trials within a group with the higher evidence level had greater neural activity than those within all the groups having lower evidence (Fig. 2*b*; see Materials and Methods).

Moreover, to determine whether or not a neuron significantly encoded choice and evidence, we measured decoding performance at the chance level by randomizing the trial labels (Fig. 2*b*). The selective neurons (Fig. 2*c*,*d*) were further visually inspected to exclude those without a ramping-like firing rate activity. The results revealed that the surviving selective neurons have DDM-like firing rate activity (Fig. 2*a*; Extended Data Fig. 2-1*a*) and are distributed across the brain regions (Fig. 2*e*; Extended Data Fig. 2-1*b*,*c*). Most DDM-like neurons were found in the frontal (MOs, PL, ACA, ILA, and ORB) and midbrain (MRN, SNr, SCm, and SCs) regions. A lower percentage of these neurons were located in the striatum (CP and ACB) and visual pathway (VISam, VISI, and VISp), thalamus (VPL, VPM, LP, PO, LD), and MOpSSp (Extended Data Fig. 2-1*b*,*c*). Some of the discovered DDM-like subareas within the frontal, striatum, and visual regions were consistent with the previous studies on the neural basis of evidence accumulation in rodents (Hanks et al., 2015; Scott et al., 2017; Yartsev et al., 2018). A single hemisphere contained neurons with both ipsilateral and contralateral choice preferences in most grouped regions (Fig. 2*e*), consistent with the previous studies (Scott et al., 2017). The frontal region was mostly bilateral since the number of the ipsilateral and contralateral DDM-like neurons was similar. In contrast, other brain regions were mostly unilateral.

### Multiple accumulation mechanisms across the brain

Previous studies on the evidence accumulation process proposed different network architectures for evidence integration including single and dual accumulators (Bogacz et al., 2006). Single accumulators such as the drift-diffusion model (DDM; Ratcliff, 1978) and the ramping model (Latimer et al., 2015; Zoltowski et al., 2019) contain one decision variable accumulating the relative evidence (difference between the two input streams) toward one of the decision boundaries. Dual accumulators are other accumulation mechanisms with separate accumulators for each choice option that integrate the input streams independently (Ditterich et al., 2003; Mazurek et al., 2003) or with mutual inhibitory connections (Usher and McClelland, 2001; X.J. Wang, 2002; Machens et al., 2005; Wong and Wang, 2006; Wong et al., 2007). In these accumulation mechanisms, an option is chosen when the integrator associated with that option reaches the decision boundary sooner than the others (Bogacz et al., 2006).

To investigate whether the DDM-like neurons across the mouse brain integrate evidence through a single or dual accumulation mechanism, we used a general framework for the evidence accumulation modeling based on the recurrent switching linear dynamical system (rSLDS; Zoltowski et al., 2020; Fig. 3). Using rSLDS, the high-dimensional population neural activity can be described as the dynamics of a few continuous latent variables in a low-dimensional state space, evolving through time according to state-dependent dynamic models (Fig. 3*a*). The rSLDS was reformulated to implement the single, independent race, and dependent race accumulation mechanisms (Fig. 3*b*) by considering the accumulators as the continuous latent variables of the model (Fig. 3*c*; Zoltowski et al., 2020).

We first generated subpopulations of neural activity by resampling the neurons within each region (see Materials and Methods). Several bilateral (including neurons with contralateral and ipsilateral choice preference) and unilateral (including neurons with contralateral choice preference) subpopulations were generated during the resampling process (Fig. 4*c*; see Materials and Methods). We fit the single and race accumulators to the bilateral subpopulations since these subpopulations contain neurons with both contralateral and ipsilateral choice preferences. On the other hand, the unilateral subpopulations contain neurons with only contralateral choice preference, so we only fit the single accumulator to them. The best initial parameters of the dynamic models were selected through a greedy search approach (see Materials and Methods).

Since we modeled the evidence accumulation phase of the decision-making process, we excluded the neural activity during the visual encoding phase from the accumulator modeling by estimating the accumulation latency using the auROC metric (see Materials and Methods). The evolution of the single and independent race variables in sample trials is illustrated in Figure 4*a*. As shown in this figure, the discrete state switches to the wheel movement state when the continuous variables reach the decision boundary.

We computed the explained variance (*R*^2^) of the models in both bilateral and unilateral subpopulations (Fig. 4*f*; Extended Data Fig. 4-1). Moreover, the best model for bilateral subpopulations was determined using the AIC difference approach (Fig. 4*d*; see Materials and Methods). The number of preferred models in the regions for both unilateral and bilateral subpopulations is depicted in Figure 4*e*. We did not observe a significant difference between the number of single and race accumulators for bilateral subpopulations (Extended Data Fig. 4-1*b*). This may be because of the scarcity of bilateral subpopulations within most of the regions. Therefore, we also compared the number of single and race accumulators among total subpopulations assuming that unilateral subpopulations could just prefer single accumulators (Extended Data Fig. 4-1*c*). As you can see in this figure, the thalamus, visual, and midbrain areas, which are more unilateral, prefer the single accumulator significantly more than race accumulators (sign test, *p*-value < 0.001). We also observed a significant difference between the number of single and race accumulators within the frontal region (sign test, *p*-value < 0.001), suggesting that this area prefers the single accumulator more than the race ones.

### Distributed evidence accumulation over multiple timescales

**Figure 5.**
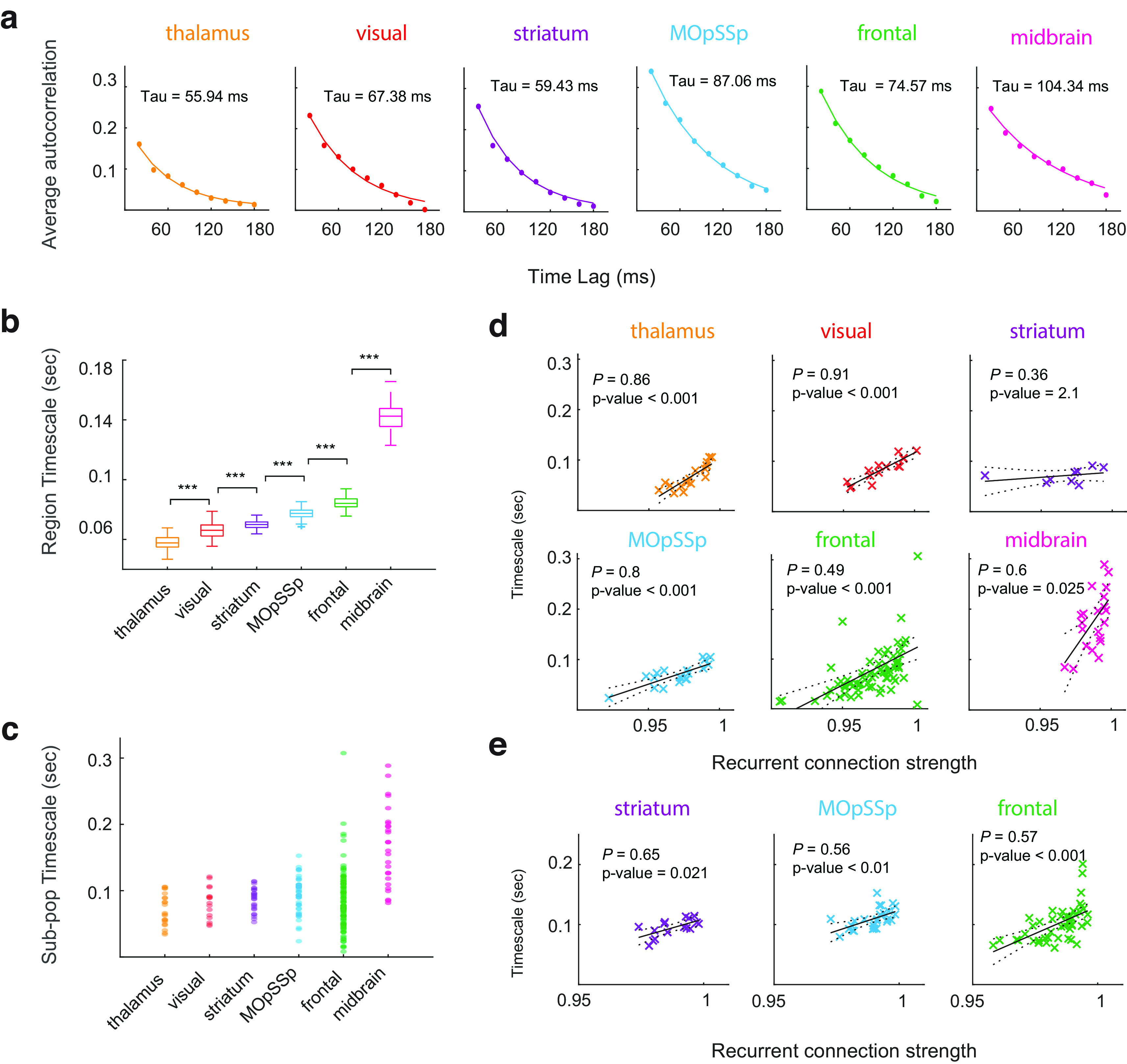
Distribution of the integration timescale across the brain. ***a***, Autocorrelation structure of a simulated subpopulation of neurons is described using the exponential decay function. ***b***, Hierarchical organization of the brain areas in terms of the integration timescale. Timescales were estimated using the combined autocorrelations of the sampled subpopulations during a 100-times bootstrapping process. Marker *** indicates the *p*-value < 0.001 in the Wilcoxon rank-sum test corrected for multiple comparisons. ***c***, Heterogeneity of the subpopulations’ timescale within each brain area. ***d***, Pearson’s correlation between the recurrent connection strength and the integration timescale of single accumulators within each brain area. *p*-values were corrected by the Bonferroni multiple comparison correction. ***e***, Pearson’s correlation between the average recurrent connection strength of the left and right accumulator variables and the integration timescale of race accumulators within each brain area. *p*-values were corrected by the Bonferroni multiple comparison correction.

The distributed coding of evidence accumulation across the brain suggests that the accumulation process is happening over multiple timescales, which can be organized hierarchically across the brain (Murray et al., 2014; Pinto et al., 2022). The ability of the brain to function in different timescales stems from the heterogeneity of local microcircuits and their long-range connectivity (Chaudhuri et al., 2015). Here, we examined whether the single and race accumulator models across the brain have distinct properties in terms of the integration timescale. Accordingly, we simulated neurons’ activity within each subpopulation using the preferred accumulator model. The integration timescale was estimated using the combined autocorrelation structure of the simulated neurons’ activity at both the local subpopulation and global population levels within the brain regions (see Materials and Methods; Fig. 5*a*,*b*). The estimated population-level timescale displayed a hierarchical organization across the brain, starting from the visual to the frontal in the cortical regions and the thalamus to the midbrain in the subcortical ones (Fig. 5*b*), which is consistent with previous studies (Chaudhuri et al., 2015; Pinto et al., 2022). The resulting hierarchy demonstrates that thalamic and visual areas integrate the information in a shorter timescale than the midbrain and frontal regions.

In addition to the hierarchical organization of integration timescale, we also observed a heterogeneity of timescales within each brain area (Fig. 5*c*). We hypothesized the observed diversity of integration timescales could reflect the differences in the accumulator microcircuits. To address this hypothesis, we explored the association between the integration timescale and the recurrent connection strength of the accumulators within each brain area using Pearson’s correlation. The results demonstrated that the recurrent connection strengths of single accumulators were significantly correlated with the integration timescales in most of the regions (Fig. 5*d*). We also examined Pearson’s correlation on the bilateral subpopulations preferring race accumulators by excluding regions with insufficient samples (<10 subpopulations; Fig. 5*e*). The results revealed that the average recurrent connection strengths of the left ($x_{L}$) and right ($x_{R}$) accumulators in the race microcircuits (Fig. 3*b*) were significantly correlated with the integration timescales in all the remaining regions. Our findings support the hypothesis that microcircuits with longer integration timescales have larger recurrent connection strength, which is in line with the previous studies (Chaudhuri et al., 2015).

## Discussion

Although previous studies on perceptual decision-making revealed the distribution of decision coding in the mouse brain (Steinmetz et al., 2019), the contribution of these neurons to the evidence accumulation process and the underlying accumulation mechanism remain unclear. Using brain-wide electrophysiological recording in mice (Steinmetz et al., 2019), we showed that evidence accumulation during perceptual decision-making is a distributed process across the brain. We found different cortical and subcortical areas, i.e., visual and frontal cortices, MOp, striatum, midbrain, and thalamus, contain neurons with drift-diffusion model-like (i.e., evidence-sensitive ramping firing rate) activity. We showed that these regions consist of subpopulations that accumulate evidence through both single and race accumulation mechanisms. We further characterized the accumulation process in terms of the integration timescale. Our findings revealed a hierarchical organization of timescales across the brain, suggesting the existence of evidence accumulation over multiple timescales. In addition, we observed a heterogeneity of timescales within the brain regions, reflecting the diversity of the accumulator’s recurrent connection strength.

The identified brain regions in this study are consistent with and complement the existing findings on the neural substrates of evidence accumulation. Prior work has demonstrated the contribution of a subset of these areas, i.e., PPC (Shadlen and Newsome, 2001; Roitman and Shadlen, 2002), FEF (Kim and Shadlen, 1999; Ding and Gold, 2012), striatum (Ding and Gold, 2010), superior colliculus (Horwitz and Newsome, 1999), and FOF (Hanks et al., 2015) in the evidence accumulation process.

The neurons with DDM-like firing rate activity across the brain could integrate the information through single or dual accumulation mechanisms (Bogacz et al., 2006). However, the dual accumulator needs the neural populations supporting each choice alternative. The brain regions we examined contain neurons with both contralateral and ipsilateral choice preferences in the left hemisphere, which were mostly observed in the frontal area. The bilateral behavior of the regions suggested the existence of a dual accumulation mechanism within a single hemisphere, consistent with the previous studies (Ratcliff et al., 2007; Wong et al., 2007; Mante et al., 2013). We tried to investigate whether DDM-like neurons in the brain were best represented using single or dual accumulators.

Our results revealed that bilateral subpopulations within the striatum and MOpSSp strongly prefer race accumulators more than single ones. However, exploring the accumulator preferences among the combined unilateral and bilateral subpopulations demonstrated that the visual, thalamus, and midbrain regions strongly prefer the single accumulator. This may be because of the unilateral nature of these brain regions. However, despite the bilateral nature of the frontal area, the number of subpopulations with single accumulation preferences is higher than the ones preferring dual accumulators. This may be because of the single-hemisphere neural recording.

We sought to address whether the distributed nature of evidence accumulation processes was related to how neurons in different brain regions represent information at different timescales. The estimated accumulator’s integration timescale at the population level revealed hierarchical organization across the brain regions. According to this hierarchy, the integration timescale increases from visual to frontal in the cortical regions and from the thalamus to the midbrain in the subcortical ones, consistent with the previous studies (Honey et al., 2012; Chaudhuri et al., 2015; Pinto et al., 2022). Our findings lend further support to previous claims that evidence accumulation is happening over multiple timescales, and different brain areas in humans, primates, and rodents display a hierarchical organization in terms of their timescale (Honey et al., 2012; Murray et al., 2014; Chaudhuri et al., 2015; Demirtaş et al., 2019; Gao et al., 2020; Rossi-Pool et al., 2021; Pinto et al., 2022; Imani et al., 2023). We extend this literature (e.g., for most recent findings using calcium imaging data in cortical regions, see Pinto et al., 2022) by providing evidence from the analysis of electrophysiological data across the whole mouse brain. This hierarchical organization could be an essential component of the distributed evidence accumulation process across the brain (Pinto et al., 2022), which may be because of the variability in the level of recurrent excitation connections within areas (Chen et al., 2015; Gao et al., 2020), and their long-range connectivity profile (Chaudhuri et al., 2015). The hierarchical organization of the brain areas in terms of the integration timescale also suggests that the inactivation of brain areas across the cortical hierarchy could affect the performance of the decision-making process at different timescales (Zatka-Haas et al., 2021; Pinto et al., 2022). In addition to the variability of timescale across the brain, we observed heterogeneity of timescale within each brain area. Our findings suggest that this heterogeneity may arise from the variation in the local accumulation microcircuits. Such that, accumulators with longer integration timescales have higher recurrent connection strength, which is consistent with the previous studies (Chaudhuri et al., 2015).

In summary, we have investigated the neural correlate of evidence accumulation across the brain. We identified that DDM-like neurons are distributed across the brain, which can integrate information through single or dual accumulation mechanisms. These accumulator circuits were characterized using distinct integration timescales which were organized hierarchically across the brain. Our findings support the hypothesis that evidence accumulation is a distributed process over multiple timescales. Moreover, we observed a heterogeneity of integration timescales within each brain area suggesting a diversity of accumulator microcircuit parameters.

## Synthesis

Reviewing Editor: Laura Bradfield, University of Technology Sydney Decisions are customarily a result of the Reviewing Editor and the peer reviewers coming together and discussing their recommendations until a consensus is reached. When revisions are invited, a fact-based synthesis statement explaining their decision and outlining what is needed to prepare a revision will be listed below. The following reviewer(s) agreed to reveal their identity: NONE.

The reviewers were overall positive in response to this manuscript and thought it a useful addition to the literature. There are a few comments, particularly by Reviewer #2, that need to be addressed upon revision.

Reviewer #1

The current manuscript investigates the brain-wide neural basis of evidence accumulation using open-source data from a recently published large-scale electrophysiological dataset of mice performing a perceptual decision-making process. The authors sought to determine the distribution of DDM-like neurons and underlying mechanisms (single/dual neuron and microcircuit accumulators) across the brain. They indeed show that evidence accumulation during a perceptual decision-making task is distributed across most of the recorded brain regions. Importantly, they found that the existence of multiple accumulation mechanisms (race and single accumulators) and that different brain regions accumulate evidence on different time-scales.

I have no major concerns regarding this manuscript. It is a well-written manuscript that uses a large open-source dataset to ask and answer an empirical question: where and how does evidence accumulation occur in the brain? In my opinion, the data presented sufficiently supports the author’s claims that evidence accumulation is a distributed process that works via multiple mechanisms. The computational details, though at time dense, are elegantly explained throughout the Results section and strongly detailed in the Methods.

The provided source code is a relatively easy to follow, though better comments throughout would improve reproducibility. My only comment is that I would suggest providing a detailed workflow or order of operation for the source code in the eventual Github repository.

Reviewer #2

Authors clearly addressed the hypotheses for three models of accumulators based on the rSLDS framwork in mice two-alternative unforced choice task. Since data in this manuscript were used from a public dataset, authors also demonstrated how they pre-processed the large-scale data with standard methods (e.g. dPCA and auROC) and what criterions they set for including the subsets of neurons in certain analyses. The results showed that different areas in mice brains had their own preferred accumulation processes mainly from one of three models. According to the previous evidences about cortical hierarchy, authors claimed that the accumulation processes across brain areas had their own heterogeneity of integration timescale, suggesting of noisy evidences accumulated in perceptual decision-making task were encoded and distributed across brain areas and worked in a manner of hierarchical way. The results showed in this manuscript are straightforward. I don’t have many major comments for it. But, there are minor comments below and it would be great if authors could provide more details for clarification.

Major comments:

Please describe how many neurons in each area were included in data analyses. This will be easier to get the ideas about the percentage of selective neurons in figure 2E and 4E. If possible, it might be more clearer if the neuron number could be added and combined with Table 1.

Minor comments:

1) According to the major comment, please add number of neurons/data points in each area in figure 2C and 2D, and all boxplots if possible.

2) In terms of the brain area abbreviations, table 1 and line 147-150 mentioned several areas were included in this manuscript but there is no original names around table 1 or in Methods. Please consider the diversity of naming system in different species and add the original names at the appropriate spaces.

3) What does the unit of mean firing rates use in this manuscript? Line 449-450 mentioned that the neurons with mean firing rates lower than 1Hz were excluded for dPCA analysis. If the same criterion was applied for all analyses, why 4B and supp-Figure 3A showed the firing rates lower than 1 Hz?

4) In terms of Figure 4D, I do understand that using AIC difference approach is a standard measurement for model comparison. But, it’s difficult to get the ideas in figure 4D. Could you elaborate it in either main text or Methods?

5) Line 226: typo ‘rece’.

6) ‘Figure 4e’ was not mentioned in the main text, but I guess it might be fit in line 230 according to the description. Also, I am confused about the values of “Subpopulation(%)” in y-axis. For example, does ‘0.8’ mean 0.8% or 80%? It’s hardly to estimate if there is no total number of neurons provided in the manuscript.

7) References inserted in the main text should be double-checked again due to the inadequate format, e.g. (Steinmetz et al., 2019) in line 344.

8) Line 581: ‘ut’ might be a typo since it is inconsistent with equation 10.

9) Line 642: ‘fne’ might be a typo since it is inconsistent with equation 16.

## Author Response

Dear Dr. Bradfield,

Thank you very much for giving us the opportunity to edit and resubmit our paper with the number ‘eN-NWR-0282-23R1’ to the eNeuro journal. We appreciate the time and effort that you and the reviewers have dedicated to providing your valuable feedback on our manuscript. We have studied the reviewer’s comments carefully and modified our manuscript to reflect their suggestions. The modified sections were highlighted in blue in the revised version of the manuscript. After these careful revisions, we hope the manuscript satisfies your high standards and requirements. The authors welcome further constructive comments.

Sincerely,

Responds to the reviewer’s comments:

Reviewer #1

Comment 1: The provided source code is a relatively easy to follow, though better comments throughout would improve reproducibility. My only comment is that I would suggest providing a detailed workflow or order of operation for the source code in the eventual Github repository.

Response: Thanks a lot for your insightful suggestion. We provided a file (README.md) containing the pipeline of the project in the GitHub repository. We will also add appropriate comments in the scripts gradually to improve the reproducibility of the program.

Reviewer #2

Comment 1: Please describe how many neurons in each area were included in data analyses. This will be easier to get the ideas about the percentage of selective neurons in figure 2E and 4E. If possible, it might be more clearer if the neuron number could be added and combined with Table 1.

Response: Thank you for raising this important point. We agree with your comment and changed Figure 2e and Figure 2-1b and the corresponding caption (line 780) to mention the number of DDM-like neurons within each brain region. However, Figure 4e displays the percentage of the sub-populations (not the neurons) preferring each accumulator model. The exact number of sub-populations is also depicted in Figure 4c.

Comment 2: According to the major comment, please add number of neurons/data points in each area in figure 2C and 2D, and all boxplots if possible.

Response: Thanks for your consideration. The number of neurons within boxplots for each brain region in Figure 2c and 2d is the same as in Figure 2e. According to comment 1, we included the number of neurons both in the y-axis label and the caption of Figure 2e.

Comment 3: In terms of the brain area abbreviations, table 1 and line 147-150 mentioned several areas were included in this manuscript but there is no original names around table 1 or in Methods. Please consider the diversity of naming system in different species and add the original names at the appropriate spaces.

Response: Thanks a lot for pointing this out. We add the original name and the corresponding acronym for each brain area in the extended Table 1-1. The full names and the acronyms are taken from the Allen Common Coordinate Framework (CCF).

Comment 4: What does the unit of mean firing rates use in this manuscript? Line 449-450 mentioned that the neurons with mean firing rates lower than 1Hz were excluded for dPCA analysis. If the same criterion was applied for all analyses, why 4B and supp-Figure 3A showed the firing rates lower than 1 Hz?

Response: Thanks a lot for your consideration. The criteria for excluding neurons from dPCA analysis was the average total number of spikes within the reaction time boundaries across trials. Since this boundary is not a fixed length and is lower than 1 second, we can not use Hz (number of samples per second) as a unit. We modified the manuscript and removed Hz from the text. However, the firing rate timecourses in the figures were computed in a different way using the half-gaussian kernel with a standard deviation of 0.02s. Therefore, the average firing rate per time point could be lower than 1.

Comment 5: In terms of Figure 4D, I do understand that using AIC difference approach is a standard measurement for model comparison. But, it’s difficult to get the ideas in figure 4D. Could you elaborate it in either main text or Methods?

Response: Thanks a lot for pointing this out. To visualize the preferred models, we computed the paired AIC differences (Δ_si=〖AIC〗_single-〖AIC〗_(independent race), Δ_sd=〖AIC〗_single-〖AIC〗_(dependent race), Δ_id=〖AIC〗_(independent race)-〖AIC〗_(dependent race)). The preferred model is the one with a lower AIC value than others. Therefore, subpopulations preferring the single model have negative AIC differences Δ_si and Δ_sd and independent race models have positive Δ_si and negative Δ_id values. Similarly, subpopulations with a dependent race preference have positive AIC differences Δ_sd and Δ_id. We also modified the manuscript to describe it in lines 388 to 393.

Comment 6: Line 226: typo ‘rece’.

Response: Thanks a lot for your comment. We corrected it in the manuscript.

Comment 7: ‘Figure 4e’ was not mentioned in the main text, but I guess it might be fit in line 230 according to the description. Also, I am confused about the values of “Subpopulation(%)” in y-axis. For example, does ‘0.8’ mean 0.8% or 80%? It’s hardly to estimate if there is no total number of neurons provided in the manuscript.

Response: Thanks for your consideration. We modified the manuscript (lines 496-497) and mentioned Figure 4e and Figure 4-1b in the text. We also changed the y-axis label in Figure 4e, Figure 4-1b, and Figure 4-1c to clear up ambiguity.

Comment 8: References inserted in the main text should be double-checked again due to the inadequate format, e.g. (Steinmetz et al., 2019) in line 344.

Response: Thanks for your comment. The references were included according to the APA 7th format. We double-checked the references. However, according to this format, all references look appropriate.

Comment 9: Line 581: ‘ut’ might be a typo since it is inconsistent with equation 10. Line 642: ‘fne’ might be a typo since it is inconsistent with equation 16.

Response: Thanks a lot for your consideration. We corrected these typos in the text (lines 319 and 377).
