# Supplementary material for: Distributed Coding of Evidence Accumulation across the Mouse Brain Using Microcircuits with a Diversity of Timescales
Source: eNeuro. 2023 Nov 2;10(11):ENEURO.0282-23.2023. doi: 10.1523/ENEURO.0282-23.2023 (PMC10626503; doi:10.1523/ENEURO.0282-23.2023)
Supplement: Extended Data Table 1-1 — The full name and acronym of brain regions within each group of areas according to the Allen CCF. Download Table 1-1, DOC file. [file enu-eN-NWR-0282-23-s01.doc]

**Table 1-1** The full name and acronym of brain regions within each group of areas according to the Allen CCF

| **Area Name** | **Acronym** | **Group Name** | **Area Name** | **Acronym** | **Group Name** |
| --- | --- | --- | --- | --- | --- |
| Postsubiculum | POST | Hippocampus | Laterointermediate visual area | VISl | Visual |
| Subiculum | SUB | Hippocampus | Anterior visual area | VISa | Visual |
| Dentate gyrus | DG | Hippocampus | Caudoputamen | CP | Striatum |
| Field CA1 | CA1 | Hippocampus | Globus pallidus, external segment | GPe | Striatum |
| Field CA3 | CA3 | Hippocampus | Nucleus accumbens | ACB | Striatum |
| Lateral posterior nucleus of the thalamus | LP | Thalamus | Lateral septal nucleus | LS | Striatum |
| Lateral dorsal nucleus of thalamus | LD | Thalamus | Secondary motor area | MOs | Frontal |
| Reticular nucleus of the thalamus | RT | Thalamus | Anterior cingulate area | ACA | Frontal |
| Mediodorsal nucleus of thalamus | MD | Thalamus | Prelimbic area | PL | Frontal |
| Medial geniculate complex | MG | Thalamus | Infralimbic area | ILA | Frontal |
| Dorsal part of the lateral geniculate complex | LGd | Thalamus | Orbital area | ORB | Frontal |
| Ventral posteromedial nucleus of the thalamus | VPM | Thalamus | Primary motor area | MOp | MOpSSp |
| Ventral posterolateral nucleus of the thalamus | VPL | Thalamus | Primary somatosensory area | SSp | MOpSSp |
| Posterior complex of the thalamus | PO | Thalamus | Midbrain reticular nucleus | MRN | Midbrain |
| Posterior limiting nucleus of the thalamus | POL | Visual | Superior colliculus motor related | SCm | Midbrain |
| Primary visual area | VISp | Visual | Superior colliculus sensory related | SCs | Midbrain |
| Rostrolateral visual area | VISrl | Visual | Anterior pretectal nucleus | APN | Midbrain |
| Anteromedial visual area | VISam | Visual | Periaqueductal gray | PAG | Midbrain |
| Posteromedial visual area | VISpm | Visual | Substantia nigra reticular part | SNr | Midbrain |
